# Management of sensing issues with a subcutaneous implantable cardioverter-defibrillator in a patient with Brugada syndrome: A case report

**DOI:** 10.1016/j.hrcr.2023.09.009

**Published:** 2023-09-16

**Authors:** Satoshi Kawada, Nobuhiro Nishii, Tomofumi Mizuno, Masakazu Miyamoto, Koji Nakagawa, Hiroshi Morita

**Affiliations:** ∗ Department of Cardiovascular Medicine, Okayama University Graduate School of Medicine, Dentistry and Pharmaceutical Sciences, Okayama, Japan; † Department of Cardiovascular Therapeutics, Okayama University Graduate School of Medicine, Dentistry and Pharmaceutical Sciences, Okayama, Japan

**Keywords:** S-ICD, Lead fracture, Lead repositioning, SMART Pass, Lead model 3401

**Key Teaching Points**

• We report a case of sensing issues managed with a subcutaneous implantable cardioverter-defibrillator (S-ICD) in Brugada syndrome with decreased QRS amplitude and QRS/T ratio over time.
• A microfracture of the model 3401 S-ICD lead, which produced oversensing of nonphysiological electrical noise artifacts, was identified because of inappropriate ventricular fibrillation episodes.
• Inappropriate shock can be prevented through remote monitoring.

## Introduction

Subcutaneous implantable cardioverter-defibrillators (S-ICDs) are an alternative to transvenous ICD (TV-ICD) for the prevention of sudden cardiac death.^1^ S-ICDs have the additional advantage of avoiding complications related to TV-ICD, such as infective endocarditis, cardiac perforation, venous occlusion, and lead dysfunction.^2^ S-ICD is a good candidate for younger patients without organic heart disease, such as Brugada syndrome (BrS), who do not require ventricular pacing. However, the morphology-based sensing system of the S-ICD is often vulnerable to oversensing. In patients with BrS, the most common reason of inappropriate sensing of S-ICD is T-wave oversensing. Other causes include electric noise caused by trapped air escaping from the device header and paroxysmal supraventricular tachycardia.^3^ In a clinical setting, managing sensing issues over implant time is essential, particularly in BrS, in which lead microfracture is part of the differential diagnosis. The model 3401 S-ICD lead was approved in September 2012 in the United States and made available in Japan between February 2016 and December 2017. According to the manufacturer’s most recent product performance report (February 20, 2023), 43,000 leads were distributed worldwide. The overall incidence of lead failure was 0.19%/year.^4^ However, details of fracture of 3401 leads have not been reported because of the low incidence rate, and not all fractured leads have been returned for the analysis. Herein, we report a case of BrS in a man with an implanted S-ICD, revealing an S-ICD 3401 lead fracture after sensing electrogram noise.

## Case report

The patient was initially referred to our hospital at the age of 42 years with electrocardiography (ECG) findings consistent with BrS and a family history of sudden cardiac death. His older brother had died suddenly after drinking alcohol. Ventricular fibrillation (VF) was induced by programmed electrical stimulation, and *SCN5A* gene test results were positive. The patient was asymptomatic but at risk of VF; therefore, we implanted a dual-chamber ICD (DDD, 50 ppm). During the follow-up, the patient developed persistent gram-negative *Salmonella bongori* bacteremia, which ultimately required removal of the entire TV-ICD system 12 years after the original implant placement. Before S-ICD implantation, screening ECG was performed, and 2 (alternate and secondary vector) of 3 vectors were available.

At the age of 54 years, the patient underwent S-ICD implantation (generator: EMBLEM model A209; subcutaneous electrode lead 3401; Boston Scientific, Marlborough, MA) with the 3-incision technique. The sensing of 2 vectors (secondary and alternate) were appropriate, and impedance values were within normal limits. Defibrillator threshold testing (DFT) was successful at 65 J. The S-ICD was programmed with tachyarrhythmic and conditional shock zones of 250 and 170 beats per minute, respectively. After 6 months from discharge, inappropriate ICD shocks were delivered while the patient was in a sauna owing to T-wave oversensing, resolved by programming vector from secondary to alternate and turning on the SMART Pass filter.

**Figure 1:**
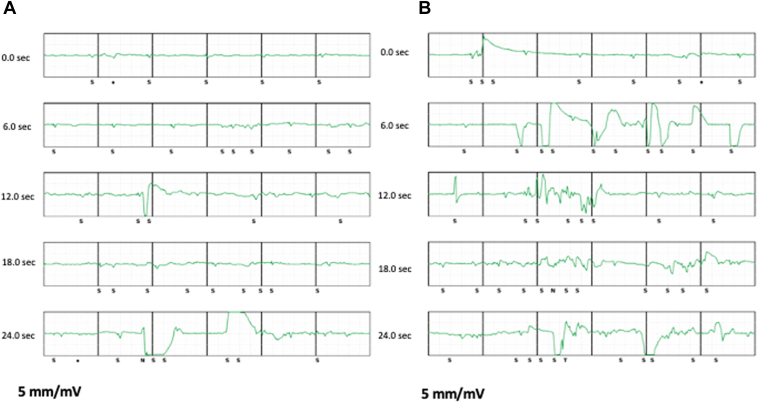
Captured subcutaneous implantable cardioverter-defibrillator electrograms. **A:** Remote alerts show inappropriate ventricular fibrillation (VF) episodes, oversensing high-amplitude nonphysiological artifacts. However, during device interrogation, the sensing electrograms are appropriate, and noise is not observed. **B:** Three months later, the patient revisited our hospital because of a red alert of inappropriate VF via remote monitoring. Sensing electrogram noise was reproduced using maneuvers.

**Figure 2:**
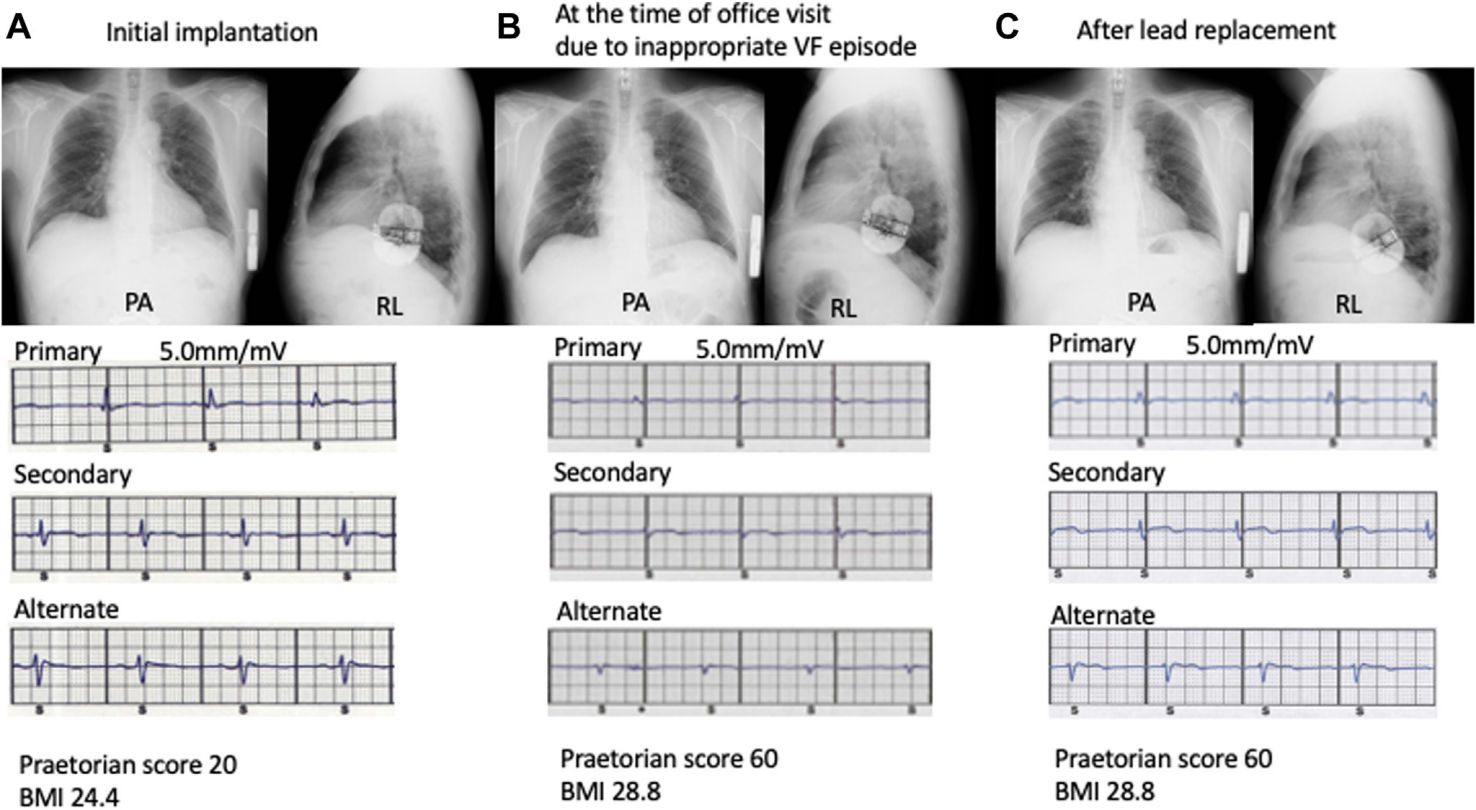
Subcutaneous implantable cardioverter-defibrillator (S-ICD) lead position and captured electrograms of all 3 vectors. Chest radiography captured electrocardiography of all 3 vectors at the time of initial S-ICD implantation (**A**), at the time of office visit because of an inappropriate ventricular fibrillation (VF) episode (**B**), and after lead replacement (**C**). The praetorian score and body mass index (BMI) were calculated. PA = posterior-anterior; RL = right-left.

**Supplemental Figure 1:**
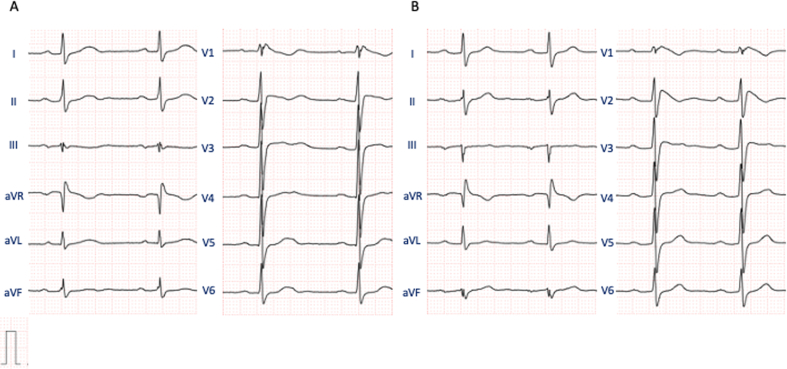
Shift in the QRS axis and QRS widening over time. Twelve-lead ECG at initial S-ICD implantation (A) and at the time of reimplantation (B). The PR interval and QRS width increased from 226 and 113 ms, respectively, to 268 and 118 ms, respectively. The QRS axis shifted from 22° to -58°. ECG, electrocardiogram; S-ICD, subcutaneous implantable cardioverter-defibrillator

After 5 years (at 59 years of age), the patient visited our hospital because of a remote alert for VF, but he was asymptomatic (Figure 1A). During the office visit, device interrogation revealed inappropriate VF episodes from oversensing of high-amplitude electrical artifacts with low QRS amplitude (0.4 mV) and QRS/T wave ratio. The vector was programmed alternate because of a decreased QRS amplitude and QRS/T wave ratio in primary and secondary vectors. Compared to the 12-lead ECG recorded at the initial S-ICD implantation, shift in the QRS axis and increasing conduction disturbance were identified (Supplemental Figure 1). The SMART Pass filter was automatically disabled owing to its low QRS amplitude. Electrode impedances were within normal limits, and chest radiography findings were normal (Figure 2B). During device interrogation, sensing electrograms were appropriate, and no noise was observed. The patient was asymptomatic and reported no history of chest trauma. We planned to turn on the SMART Pass filter and follow up closely with remote monitoring.

Three months later, he visited our hospital again because of a red alert for VF upon remote monitoring (Figure 1B). Interrogation of the S-ICD revealed 8 episodes of VF. The rhythm analysis revealed that each episode of VF had resulted from oversensing of high-amplitude electrical artifacts, which also caused a baseline drift resulting in oversensing of the isoelectric line. The QRS-wave amplitude decreased (0.4 mV), and the SMART Pass filter was turned off again. No artifacts were observed during device interrogation; however, sensing electrogram noise was reproduced along with left arm motion and mechanical stress to the lead. A lead fracture was suspected. After discussion, we decided to explant the fractured lead and replace it.

**Figure 3:**
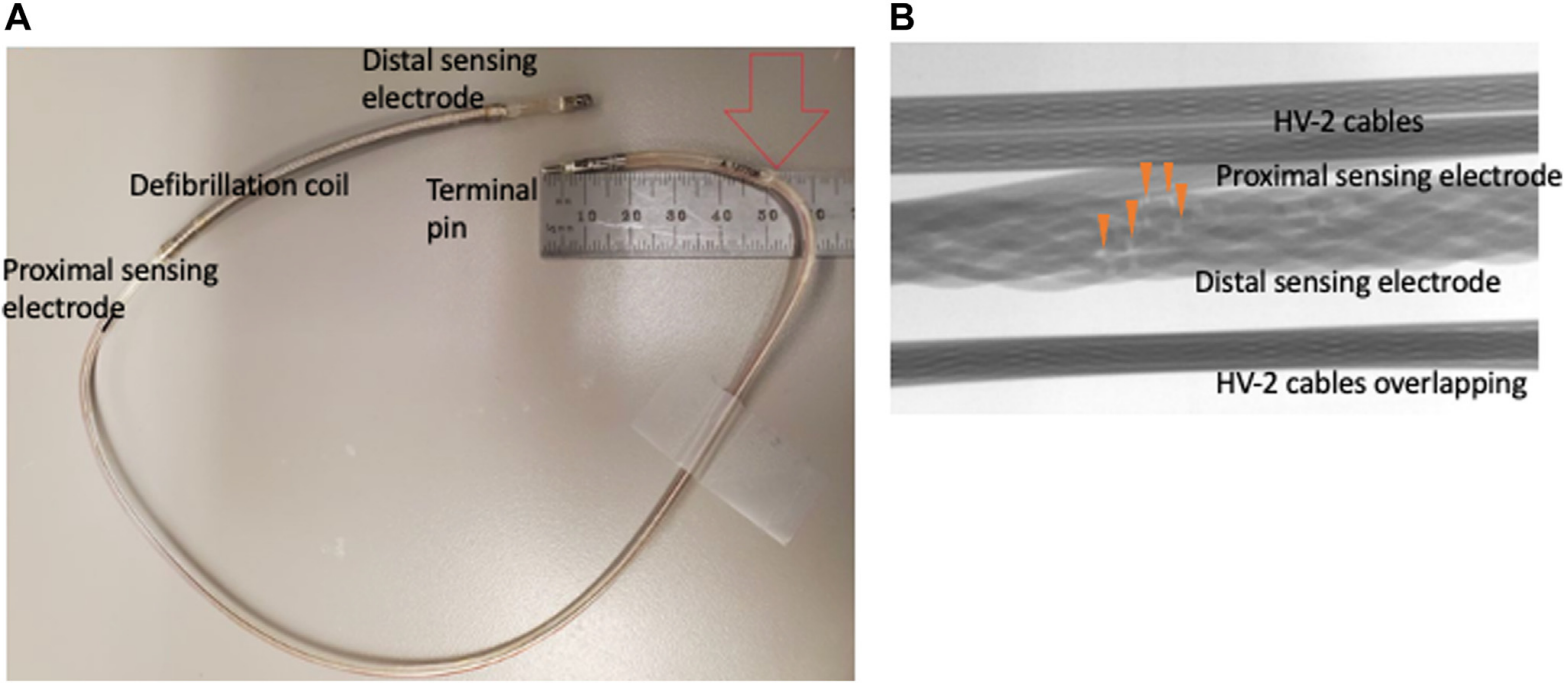
Extracted model 3401 lead showing a fracture in several filters of the distal sensing electrode. **A:** A bend is identified in the terminal pin area without indentation or surface abrasion. **B:** High-magnification radiography shows fractures in several filters of the distal sensing electrode, approximately 51 mm from the terminal pin. Yellow arrowheads indicate fractures in the electrodes.

A Byrd dilator telescoping polypropylene sheath (part number LR-PPLBES-11.5; Cook Medical, Bloomington, IN) was placed over the S-ICD lead. The polypropylene sheath rotated in a clockwise and counterclockwise fashion to mechanically disrupt fibrotic adhesions around the coil and distal tip. Finally, the S-ICD lead was completely removed. Based on a preimplantation screening, a new lead was placed slightly oblique to the sternum to achieve appropriate QRS-wave sensing (0.9 mV) and QRS/T ratio <3.5 with 2 suitable sensing vectors (Figure 2C). S-ICD was implanted with the 2-incision technique. The impedance was 60 Ω, and DFT was successful at 65 J. The fractured lead was explanted and submitted to the manufacturer for further analyses. The manufacturing analysis revealed that lead impedance between the distal and proximal electrode segments increased from 6.6 to 30 Ω with stretching. A bend was also identified in the terminal pin area, without indentation or surface abrasion (Figure 3A). A high-magnification radiograph identified a fracture in several filters of the distal sensing electrode, approximately 51 mm from the terminal pin (Figure 3B). The patient was discharged from our hospital without complications and has not experienced a remote alert or arrhythmia event for 2 years.

## Discussion

We described the management of sensing issues with an S-ICD in BrS from decreased QRS amplitude and QRS/T ratio over time. This was the first case report describing a fracture of the 3401 S-ICD lead that produced oversensing of nonphysiological electrical noise artifacts. Inappropriate ICD shocks were prevented using remote monitoring with a close follow-up.

A decreased QRS amplitude is related to poor R/S- and P-wave sensing and/or potential T-wave oversensing.^5^ The differential diagnosis for decreased R/S wave height includes lead dislodgment, QRS morphology changes owing to bundle branch block, shift in the QRS axis, subcutaneous tissue overgrowth over time, and degeneration of the myocardium, including myocardial ischemia.^6^ Subcutaneous tissue overgrowth can affect the QRS amplitude. The PRAETORIAN score helps evaluate the adequate implant position and predict DFT success of the S-ICD.^7^ The PRAETORIAN score in the present case was adequate, and both lead and generator positions seemed to be appropriate. However, it increased from 20 to 60 with increasing body mass index, which might affect the QRS amplitude over time (Figure 2). Patients with BrS with *SCN5A* mutations show progression of ECG abnormalities over time, including QRS interval and intervals between the peak and the end of the T wave.^8^ We speculated that the shift in the QRS axis and increasing conduction disturbance over time could affect the QRS amplitude (Supplemental Figure 1).

The SMART Pass filter was designed to reduce cardiac oversensing and demonstrated a 50% reduction in inappropriate therapy.^9^ It improved the eligibility of S-ICD vectors, both at rest and during exercise.^10^ Conte and colleagues^11^ demonstrated a trend toward reduction in the vector failure rate from 40% to 36% in patients with BrS when the SMART Pass filter was available. In this patient, the SMART Pass filter was automatically disabled owing to a decrease in the QRS amplitude. Monkhouse and colleagues^12^ categorized the reasons for SMART Pass filter deactivation into 3 groups: small R waves, arrhythmia with periodic axis shift, and long pause. The cause of SMART Pass filter deactivation should be investigated, and using an alert for deactivation could prevent inappropriate ICD shocks.

A small change in the lead position significantly affects the R-wave amplitude and defibrillator threshold in S-ICDs. Preimplantation screening of an S-ICD is mandatory, particularly in patients with a low-amplitude QRS complex. Sasaki and colleagues^13^ reported that changing the position of the S-ICD lead from standard left parasternal to the midline of the sternum was feasible for low QRS amplitudes and reduced muscular noise owing to myopotentials. A new lead was placed slightly oblique to the sternum based on a screening ECG before S-ICD reimplantation, achieving appropriate QRS-wave sensing in the present case. The position of the generator could improve the R-wave amplitude; however, we did not change the position of the generator, as it seemed to be adequate.^14^ Positioning of the lead to gain an appropriate QRS wave and shock impedance is feasible and would provide long-term benefits.

According to the manufacturer’s product performance report regarding model 3401, 69 malfunctioning leads have been confirmed to date.^4^ The estimated probability of survival is 97.4% at 10 years.^4^ In detail, Boston Scientific recently reported 33 electrode conductor fractures in or near the pocket and 3 weld fractures in a product performance report.^4^ The mechanism of the remaining failed leads (33 leads) was nonpatterned or unknown. In the present case, a lead fracture was suspected because of a red alert with inappropriate VF episodes because of oversensing of high-amplitude electrical artifacts with a low QRS amplitude. A high-magnification radiograph revealed fractures in several filters of the distal sensing electrode. The exact reasons for lead fracture are unknown; however, we speculate that it could have been caused by mechanical stress around the pocket.

## Conclusion

We reported a case of BrS in a man implanted with an S-ICD, revealing an S-ICD 3401 lead fracture after sensing electrogram noise.

## Disclosures

Drs Morita and Nishii are affiliated with the endowed department by Japan Medtronic Inc. Dr Nishii received remuneration for lectures from Medtronic and Boston Scientific. The remaining authors have nothing to disclose.
